# Loss of mTORC2 signaling in oligodendrocyte precursor cells delays myelination

**DOI:** 10.1371/journal.pone.0188417

**Published:** 2017-11-21

**Authors:** Mark D. Grier, Kathryn L. West, Nathaniel D. Kelm, Cary Fu, Mark D. Does, Brittany Parker, Eleanor McBrier, Andre H. Lagrange, Kevin C. Ess, Robert P. Carson

**Affiliations:** 1 Department of Pediatrics, Vanderbilt University Medical Center, Nashville, Tennessee, United States of America; 2 Department of Biomedical Engineering, Vanderbilt University, Nashville, Tennessee, United States of America; 3 Department of Neurology, Vanderbilt University Medical Center, Nashville, Tennessee, United States of America; Instituto Cajal-CSIC, SPAIN

## Abstract

Myelin abnormalities are increasingly being recognized as an important component of a number of neurologic developmental disorders. The integration of many signaling pathways and cell types are critical for correct myelinogenesis. The PI3-K and mechanistic target of rapamycin (mTOR) pathways have been found to play key roles. mTOR is found within two distinct complexes, mTORC1 and mTORC2. mTORC1 activity has been shown to play a major role during myelination, while the role of mTORC2 is not yet well understood. To determine the role of mTORC2 signaling in myelinogenesis, we generated a mouse lacking the critical mTORC2 component *Rictor* in oligodendrocyte precursors (OPCs). Targeted deletion of *Rictor* in these cells decreases and delays the expression of myelin related proteins and reduces the size of cerebral white matter tracts. This is developmentally manifest as a transient reduction in myelinated axon density and g-ratio. OPC cell number is reduced at birth without detectable change in proliferation with proportional reductions in mature oligodendrocyte number at P15. The total number of oligodendrocytes as well as extent of myelination, does improve over time. Adult conditional knock-out (CKO) animals do not demonstrate a behavioral phenotype likely due in part to preserved axonal conduction velocities. These data support and extend prior studies demonstrating an important but transient contribution of mTORC2 signaling to myelin development.

## Introduction

Myelin abnormalities are implicated in the pathogenesis of multiple neurodevelopmental disorders including Tuberous Sclerosis Complex (TSC) [1], autism spectrum disorders [2] and Angelman syndrome [3]. Elucidation of signaling pathways and cell types responsible for normal myelin development is critical to understanding disease mechanisms and developing better treatments for these disorders.

Translational studies involving multiple sclerosis (MS), a neurological disorder associated with myelin inflammation, have extended our knowledge of myelin formation and tissue response to injury. While the *in vivo* signals for oligodendrocyte precursor cell (OPC) differentiation are not precisely defined, important proteins include neuregulin, IGF-1, and Laminin-2 [4]. While contributions of other pathways are less clear, a role for PI3-K/Akt signaling has recently become more evident. The nature of this role remains unclear, however, with conflicting reports involving downstream components of the signaling pathway. Multiple studies indicate that Akt phosphorylation and subsequent mechanistic target of rapamycin complex 1 (mTORC1) activation promote myelin formation and increase myelin thickness [5,6] with loss of Akt/mTORC1 activity being associated with decreased myelination [5–7]. In contrast, Lebrun-Julien et al. [8] and our group[9] demonstrated an opposite requirement for mTORC1 with decreased myelination due to mTORC1 hyperactivity following loss of *Tsc1* in the spinal cord or *Tsc2* in the brain and spinal cord.

There is growing interest in defining the contribution of the mTORC2 complex in the central nervous system and in myelination. The mTORC2 complex is differentiated from the mTORC1 complex by inclusion of the Rictor (rapamycin insensitive companion of mTOR) protein. The biologic role of mTORC2 is still being defined with the initial studies suggesting a role in cytoskeletal support [10]. mTORC2 functions as a serine/threonine kinase with targets that include SGK1, PKCα and Akt. Full activation of Akt requires phosphorylation by both PDK1 at threonine-308 and by mTORC2 at serine-473. A role for Rictor in the central nervous system was first described in 2010, with *Rictor* ablation in neurons demonstrating decreased cortical Akt phosphorylation, deficits in sensorimotor gating and reduced prefrontal dopamine content [11]. A role for mTORC2 in oligodendrocytes is supported by data demonstrating a mild hypomyelination following inactivation of either *Rictor* or *mTOR* in oligodendrocytes [12,13]. This is in contrast to deletion of *Raptor* (regulatory associated protein of mTOR), a critical component of the mTORC1 complex, which results in a much more severe reduction in myelin related proteins.

In TSC patients and in TSC animal models [14], mTORC1 activity is increased due to disruption of the hamartin/tuberin heterodimer, which typically represses mTORC1 activation. Feedback inhibition due to excessive mTORC1 activity leads to phosphorylation of Rictor and reduced mTORC2 activity. We hypothesized that reduced mTORC2 activity contributes to behavioral abnormalities in TSC. Utilizing a mouse model with conditional inactivation of *Rictor* in neural progenitor cells (including oligodendrocyte precursors), we demonstrated that decreased mTORC2 activity alone contributes to TSC relevant phenotypes[15]. In addition to seizures and behavioral abnormalities following deletion of *Tsc1* or *Rictor* from neuroprogenitor cells, respectively, cortical hypomyelination was observed[14]. Based on these findings, we sought to determine the relative contribution of mTORC2 signaling to oligodendrocyte development and function following the deletion of *Rictor* in oligodendrocyte precursor cells.

In this manuscript, we describe a conditional knockout (CKO) of *Rictor* using an *Olig2-Cre* driver to investigate the role of mTORC2 signaling in oligodendrocyte precursors. Our findings in *Rictor* deficient OPCs recapitulate and extend those seen from targeting mature oligodendrocytes[13]. We demonstrate cortical and subcortical hypomyelination, with eventual normalization to levels seen in wild type littermates. Despite the clear hypomyelination phenotype, loss of *Rictor* from OPCs did not significantly alter locomotor activity or demonstrate changes in anxiety related behaviors, consistent with the preserved conduction velocity across the corpus callosum. These findings suggest a modest requirement for mTORC2 signaling during myelination by oligodendrocytes.

## Materials and methods

We generated a conditional knockout mouse strain lacking *Rictor* in oligodendrocytes by breeding mice harboring a floxed *Rictor* allele as previously described [15]. *Olig2-Cre*+ mice (Jackson Laboratory #011103, Sacramento, CA, USA) were crossed to *Rictor*^*F/F*^ mice to create *Rictor*^*F/Wt*^; *Olig2*-Cre-positive mice. *Rictor*^*F/Wt*^; *Olig2*-Cre-positive mice are bred with homozygous *Rictor*^*F/F*^ mice to create animals homozygous for the *Rictor* floxed allele (*Olig2*-*Rictor* CKO). Mice transgenic for *Nkx2*.*1*-Cre (Jackson Laboratory #008661, Sacramento, CA, USA) were bred with *Rictor*^*F/F*^ mice to create *Rictor*^*F/F*^; *Nkx2*.*1-Cre* (*Nkx2*.*1-Rictor* CKO) mice with *Rictor* inactivation in GABAergic interneuron progenitors of the medial ganglionic eminence. Genotyping was performed using PCR as previously described [15].

Mice were housed within Vanderbilt’s animal housing facilities under normal environmental conditions with a standard 12-hour light-dark cycle and unrestricted access to water and food. Mice were monitored daily and their physical appearances observed for any adverse effects (i.e. weight loss, hunched posture, scruffy appearance). Weekly weights were taken to ensure maintenance of normal food and water intake. No adverse effects pertaining to the loss of the Rictor protein from oligodendrocytes were noted and animals maintained normal physical appearance, fertility and lifespan until they were retired from breeding and/or euthanized following CO_2_ inhalation or anesthesia for tissue harvesting, methods in accordance with AVMA guidelines. All work was conducted with the approval of the Institutional Animal Care and Use Committee (IACUC) at Vanderbilt University, Nashville, Tennessee (M/12/295).

### Immunofluorescence

Brain tissues were dissected from *Rictor* CKO and littermate controls as previously described[15–17]. Briefly, animals were anesthetized with ketamine/xylazine and perfused (P5 and older) with ice-cold phosphate buffered saline (PBS) followed by ice-cold 4% paraformaldehyde (PFA) in PBS (pH 7.4). Brains were post-fixed in 4% PFA overnight and cryoprotected in 30% sucrose prior to sectioning. For immunofluorescence studies, sections were blocked with 5% goat serum and 0.1% Triton-X100 in PBS for 1 hour at room temperature. Primary antibodies were diluted in blocking solution and incubated overnight at 4°C. APC and Ki67 detection required antigen retrieval, performed as previously described [9,12]. Ki67 antigen retrieval consisted of a 1 hour incubation in 10 mM pH 6 citrate buffer, which had been heated to boiling. For APC expression, modified superblock (10% BSA, 10% FBS and 0.5% Triton X-100) was used with no additional permeabilization step. After washing with PBS, sections were probed with species appropriate secondary antibodies (anti-mouse, anti-rabbit, or anti-rat Alexa 488, 555, 647 fluorochromes, Invitrogen, Watham, MA, USA) for one hour at room temperature. Photomicrographs were obtained with an AMG Evos epifluorescence microscope (ThermoFisher, Watham, MA, USA). Image analysis was performed with ImageJ (version 1.47, National Institutes of Health, Bethesda, MA, USA) and Adobe Photoshop CS5 (Adobe Systems, San Jose, CA, USA). For APC cell counts, a defined area of the corpus callosum was identified and all APC positive cells within that specified region were counted. Results were expressed as APC positive cells/mm^2^ then normalized to control. Primary antibody dilutions: GFAP 1:1000, phospho-S6 (Ser240-244) 1:200, myelin associated glycoprotein (MAG) 1:100, PDGFRα 1:500 (all Cell Signaling, Danvers, MA, USA), Olig2 1:500 (Millipore, Temecula, CA, USA), MBP 1:200 (Abcam, Cambridge, MA, USA), Ki67 1:100 (BD Biosciences, San Jose, CA, USA) and APC 1:100 (Calbiochem, San Diego, CA, USA).

### Magnetic Resonance Imaging

MRI was performed as described previously [9,18]. Briefly, following anesthesia with ketamine/xylazine, mice underwent intracardiac perfusion with 2% paraformaldehyde/2.5% glutaraldehyde/1mM gadopentanoic acid (Gd-DTPA; Magnevist, a contrast agent) in PBS followed by post-fixing for 1 week at 4C. Brains were then washed 3–4 times over one week with 1X PBS/1mM Gd-DTPA/0.01% sodium azide to remove excess fixative and recover innate tissue properties. 3D MRI was performed on a 15.2 Tesla (T) 11-cm horizontal bore Bruker Biospec scanner (Bruker BioSpin, Billerica, MA, USA). Myelin water fraction (MWF) was derived from multiexponential T_2_ data with MWF defined as the percentage of signal with *T*_2_<17 ms [19–21]. Anatomical measurements were derived from high resolution T_1_-weighted images, 300 μm off midline for sagittal sections and at bregma -1.5 mm for coronal sections. For MWF quantitation, the mean MWF from three regions of the corpus callosum for each animal were averaged to generate a single data point per animal.

### Immunoblotting

Mice were anesthetized with isoflurane and tissues rapidly dissected on ice, flash frozen in liquid nitrogen and stored at -80°C. The underlying corpus callosum was included with the cortical tissues. Lysate preparation, SDS-PAGE, and western blotting were performed as described previously [15]. Primary antibodies: pS6 (Ser235/236), pS6 (Ser240/244), S6, pAkt (Ser473), pAkt (Thr308), Akt, pNDRG1-Thr346, NDRG1, MAG, GFAP, CNPase, and actin (Cell Signaling, 1:1000 dilution); myelin basic protein 1:1000 (rat, Abcam), PGP9.5 1:2000 (Serotec, Kidlington, UK), and actin 1:2000 (mouse, Sigma, St. Louis, MO, USA).

### Transmission Electron Microscopy (TEM)

Mouse brains were fixed as described above for MR-imaging and subsequently processed for TEM per standard protocols. Briefly, following a mid-sagittal cut, sections of the midbody of the corpus callosum from the perfusion fixed brains were sectioned in the sagittal plane and processed for EM at the Vanderbilt Cell Imaging Shared Resource Electron Microscopy facility, as previously described[9,18]. 70-80nm ultra-thin sections were collected on 300-mesh copper grids, post-stained with 2% uranyl acetate and lead citrate and imaged with a Philips/FEI Tecnai T12 electron microscope

#### Determination of axon density and calculation of G-ratios

15,000x field images were used for determination of myelin thickness and axon diameter using a semi-automated method as previously described[21]. From these values, g-ratio, the ratio of axon diameter to the myelinated axon diameter was calculated. Myelin fraction, the fraction of myelin area to total field area was likewise determined from 15,000x images. Myelinated axon density was determined from 6,500x field images. A minimum of 50 axons per animal with an n>3 animals were used per group. Histological measures were then compared between the control and CKO groups using 2-way ANOVA.

### Electrophysiology

For brain slice preparation, postnatal day (P) 32–42 mice were decapitated under anesthesia with isoflurane and brains were quickly removed and placed in ice-cold cutting solution containing sucrose (200 mM), KCl (1.9 mM), Na2HPO4 (1.2 mM), MgCl2 (6 mM), CaCl2 (0.5 mM), glucose (10 mM), and NaHCO3 (25 mM). Coronal slices (400 μm) containing the corpus callosum were obtained using a Leica VT1200S vibratome. Slices were immediately transferred to holding chambers containing warmed (30°C) artificial cerebrospinal fluid (ACSF) containing NaCl (125 mM), KCl (2.5 mM), Na2HPO4 (1.25 mM), MgCl2 (1.3 mM), CaCl2 (2.0 mM), glucose (10 mM) and NaHCO3 (25 mM). Following 30 minutes of recovery, slices were maintained at room temperature for a minimum of 1 hour before being transferred to a recording chamber. The recording chamber was perfused with oxygenated ACSF at a rate of 2.0 mL/min. Experiments were conducted at 25°C and temperature was controlled by in-line and bath heaters (Warner Instruments, Hamden, CT). Field recordings were performed using an Axon MultiClamp 700B amplifier, filtered at 2 kHz and digitized at 10 kHz using a Digidata 1440A analog to digital converter (Molecular Devices, Union City, CA). The corpus callosum was identified using a Nikon Eclipse FN1 microscope equipped with infrared-differential interference contrast (IR-DIC) imaging. To measure field potentials, glass recording microelectrodes (3 to 5 MΩ) were placed at varying distances from the stimulating electrode as described previously [22]. Conduction velocity was determined by calculation of the inverse slope of the linear regression of response times.

### Behavioral studies

All behavioral experiments were conducted in the Vanderbilt Murine Neurobehavioral Core and approved by the Vanderbilt IACUC. Animals were housed on a 12-hour light-dark cycle with *ad libitum* access to food and water. Mice were allowed to acclimate to the environment for a minimum of 7 days prior to experiments.

#### Open field

Locomotor activity in a novel environment was quantitated with a commercial activity chamber measuring 10.75 x 10.75 inches (Med Associates, St. Albans, Vermont)..within ventilated, sound-attenuating boxes as described previously[15]. Activity was monitored for 50 minutes using Activity Monitor software (Med Associates, St. Albans, Vermont).

#### Elevated zero maze

The maze apparatus was used as described previously [15]. Animals were placed in the open arm of the maze and activity was recorded for 5 minutes. Data was acquired and analyzed using the ANY-Maze video tracking software program (SDI, San Diego, California, USA).

#### Rota-rod

Balance and coordination were measured using a Rota-rod device (Ugo Basile, Varese, Italy). Animals were placed on rotating rod at 5 RPM with graded increases in rotational velocity over 5 minutes to a maximum of 49 RPM. Latency to the animals fall from the rod or to hold on and complete two complete revolutions on the rod was recorded. Animals were trained on the apparatus with 3 trials on 2 consecutive days prior to testing on day 3. The average latency to fall over 3 trials on day 3 was recorded.

#### Marble Burying

Test cages were prepared with 15 black marbles distributed evenly on top of 2.5 inches of white bedding material. One animal was placed in the center of each of the test cages for 30 minutes. If two-thirds or more of the marble was covered with bedding, it was considered buried.

#### Barnes maze

Barnes maze was used as described for analysis of spatial memory[23]. Animals were placed in the center of a white, acrylic, circular table (90 cm diameter) with 12 equally-spaced 5 cm holes located 5 cm from the edge. A black acrylic escape box (8x8x8 cm), to which the mice can gain access by way of a white acrylic ramp, was fitted under one of the holes in place of the door. Bright light was used as an aversive stimulus encouraging the mice to find the escape. Mice underwent 3 training trials daily for 3 days in which to locate the escape. A probe trial was performed on day 4 in which the opening to the escape was covered. Visual cues remained consistent during the study period. Latency to reach the escape and path length to the escape were acquired and analyzed using the ANY-Maze video tracking software program (SDI, San Diego, California, USA).

## Results

**Fig 1:**
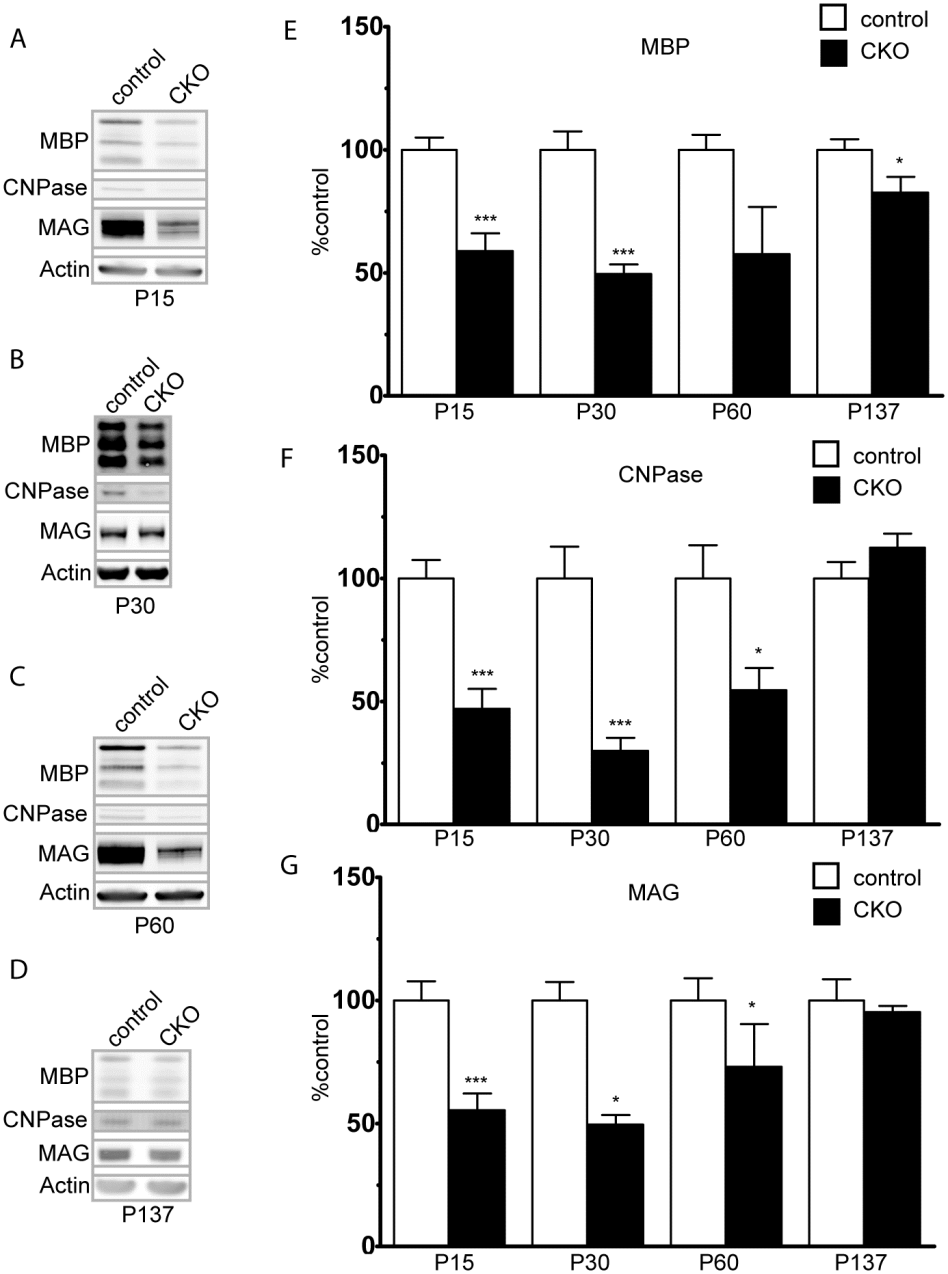
Hypomyelination secondary to Rictor loss in OPCs. Expression of the myelin associated proteins MBP, CNPase and MAG in mouse cortical protein extracts starting at P15. Data represent mean +/- SEM compared with Student’s t-test, n = 3–7 animals per group.*p<0.05, ***p<0.001.

Decreased mTORC2 activity secondary to loss of *Rictor* from oligodendrocytes has been reported to decrease expression of myelin related proteins in a mouse model targeting mature oligodendrocytes[13]. To determine if loss of mTORC2 activity from oligodendrocyte precursor cells decreases cortical myelination, cortical lysates from *Rictor* CKO mice were analyzed with immunoblotting and demonstrated a marked reduction of in the expression of myelin related proteins MBP, CNPase, and MAG, with the most pronounced abnormalities between P15 and P30 (Fig 1). The degree of hypomyelination decreased over time and by P137, both MAG and CNPase levels had normalized though MBP remained slightly, but significantly reduced. These findings are consistent with those of Bercury et al.(13), and support a role for Rictor/mTORC2 during cortical myelination.

**Fig 2:**
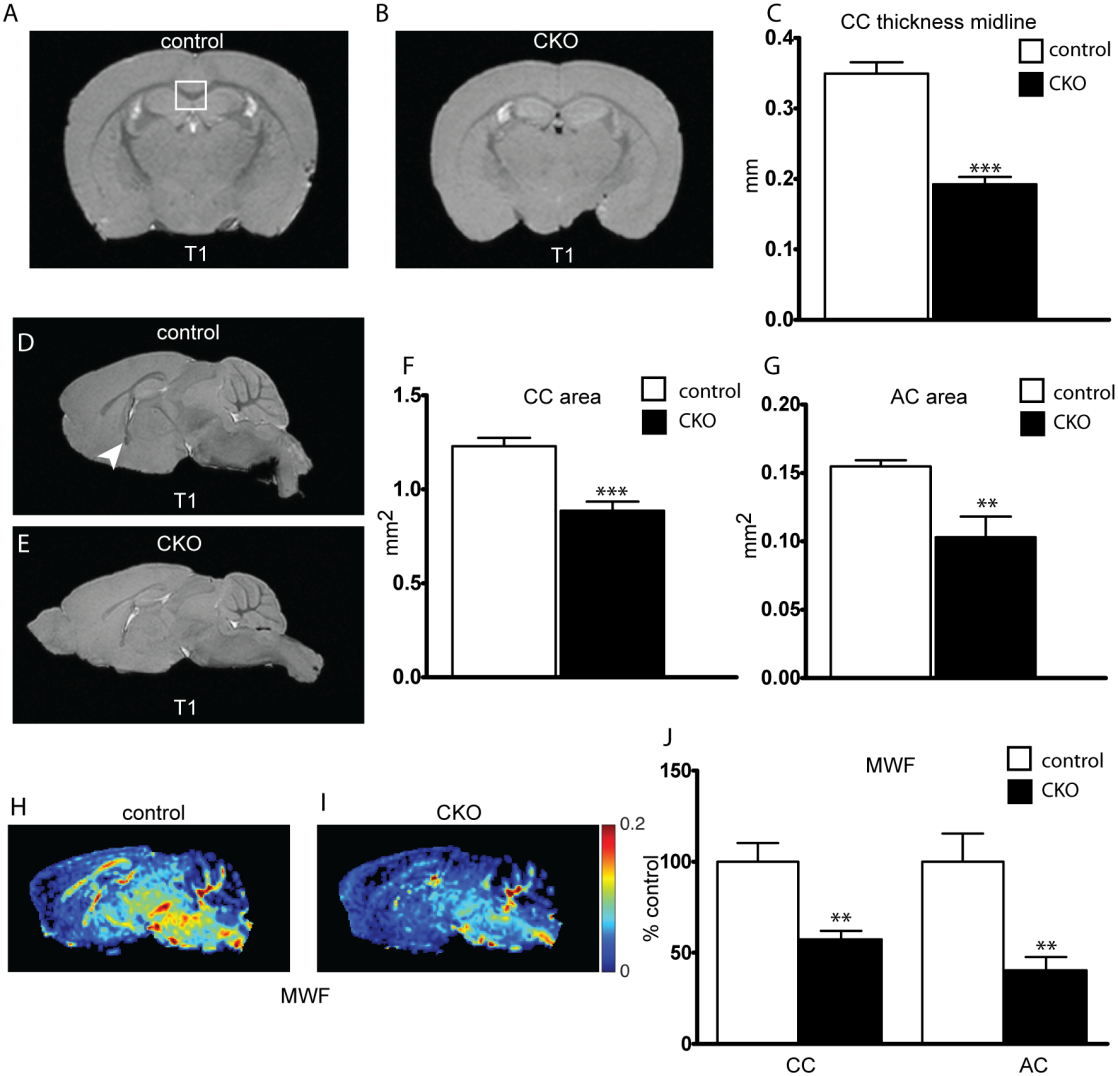
Thickness of myelinated tracks is reduced following loss of *Rictor*. Corpus callosum (CC, white box) thickness and area were determined in control and CKO mouse brains ex-vivo from T_1_-weighted MRI images obtained at 15.2T. Width determined at midline in coronal sections(A-C) and area in sagittal sections(D-F). Anterior commissure (arrow head) area was determined from sagittal sections (G). Determination of the percent of water bound between myelin bilayers, the myelin water fraction (MWF), was calculated from regions of interest in the CC and AC(H-J). Data represents mean +/- SEM compared with Student’s t-test, n = 5–7 animals per group, *p<0.05, **p<0.01, ***p<0.001.

Diffusion weighted MRI studies have demonstrated abnormalities in myelination in multiple neurodevelopmental diseases including TSC. To this effect, we used MRI to characterize structural changes in the mouse brain following loss of *Rictor*. High resolution T_1_-weighted imaging demonstrated clear reductions in size of myelinated tracts. Corpus callosum (CC) thickness, measured at the midline from coronal sections, and CC area, measured 300 μm away from midline in sagittal sections, was significantly decreased (Fig 2A–2F). A similar reduction in area was seen in the anterior commissure (Fig 2E–2G). Myelin water fraction (MWF), a measure originating from water constrained by myelin lipid bilayers, was significantly reduced in both the CC and AC (Fig 2H–2J), further supporting hypomyelination resulting from the loss of *Rictor* in OPCs.

**Fig 3:**
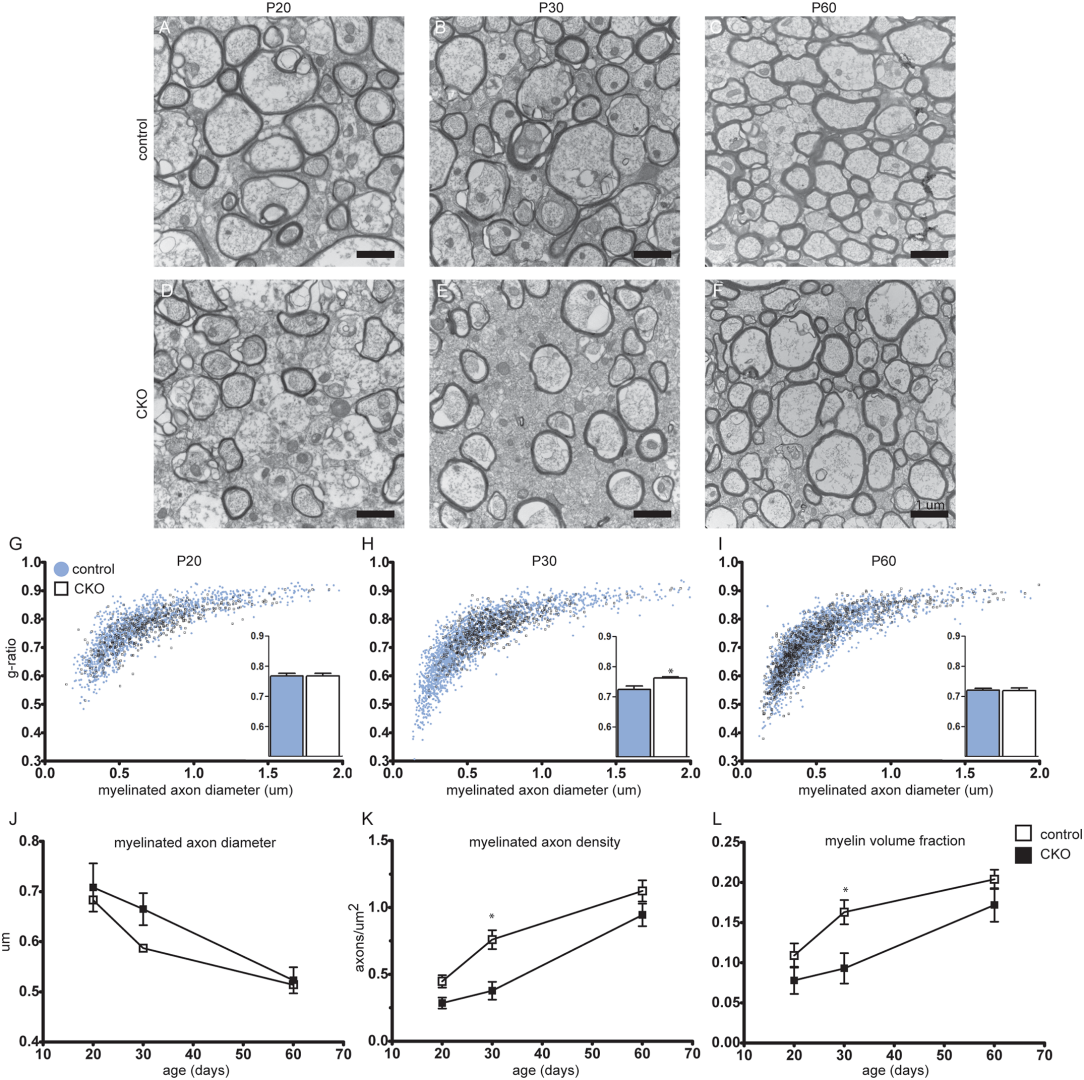
Myelinated axon density is decreased in the Rictor CKO. Sagittal sections from the mid-CC were analyzed with TEM. Representative photomicrographs at 15,000x from control (A-C) and CKO CC (D-F) at P20, 30, 60 are shown. Myelinated axon diameter was measured and g-ratio calculated from the 15,000x photomicrographs (G-J). Myelinated axon density, axons/μm^2^, was determined from 6,500x photomicrographs (K). The myelin fraction, defined as the area of myelin/area of the image was determined from 15,000x photomicrographs (L). Data represents mean +/- SEM compared with 2-way ANOVA, n = 3–6 animals per group, *p<0.05. Scale bar = 1 μm.

To define the effects of *Rictor* loss on axon ultrastructure, photomicrographs were obtained from the mid-CC of control and CKO mice at P20, P30, and P60 (Fig 3). An increase in myelinated axon density can be appreciated with age in both control and CKO animals. The g-ratio, the ratio of axon diameter to myelinated axon diameter is unchanged between control and the *Rictor CKO* at P20, though is significantly increased at P30 in the CKO. By P60, the g-ratio is again the same as the control. This closely parallels the changes in axon diameter over time, with bias towards myelination of larger diameter axons preferentially in the CKO vs. the control. The density of myelinated axons as well as the myelin fraction both increase over time, though both are significantly reduced in the CKO at P30 vs. the controls. In total, these data support a delay in myelination due not to a change in the amount of myelin per axon, but due to a temporal reduction in the density of myelinated axons.

**Fig 4:**
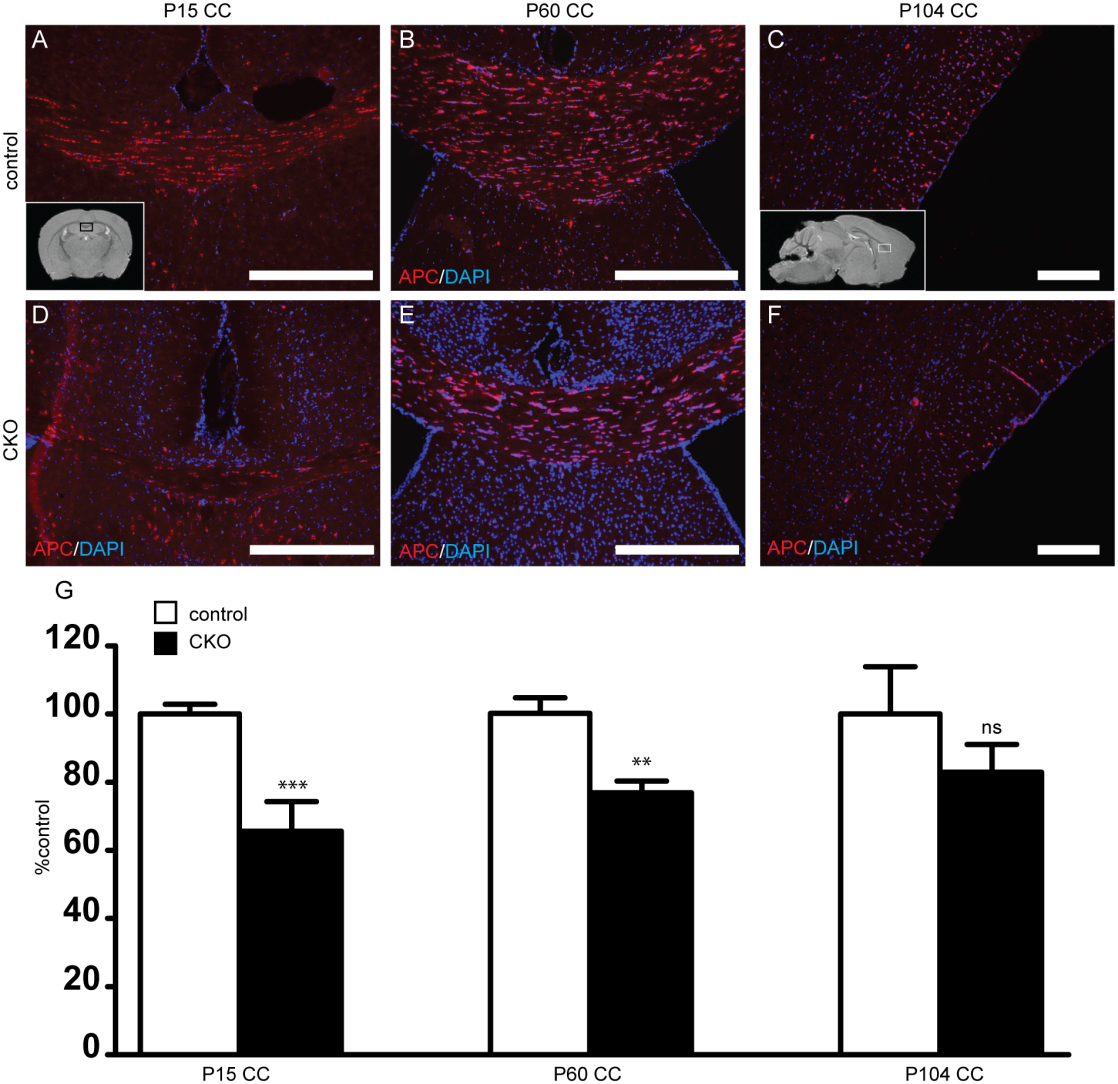
Decreased mature oligodendrocytes in the corpus callosum following loss of *Rictor*. To determine if loss of mTORC2 activity alters oligodendrocyte number, numbers of APC-positive mature oligodendrocytes were counted in the corpus callosum in P15 (A, D, G), P60 (B, E, G) and P104-P111 (C, F, G) day old mice. (Inset A, black box represents area of CC depicted on coronal sections. Inset C, box represents area of CC depicted from sagittal sections). Data represents mean +/- SEM compared with Student’s t-test, n = 4–10 per group, **p<0.01, ***p<0.001. Scale bar = 200 μm (A-F).

To determine if reduced myelination is due to a decrease in oligodendrocyte number, mature APC+ oligodendrocytes were counted in the corpus callosum and anterior commissure of P15 to P111 mice. At P15, APC positive oligodendrocyte number was decreased by 35% in *Rictor* CKOs versus control littermates (Fig 4). By P60, cell density was still reduced compared to controls, though the magnitude of the difference decreased to 23%. By P104, there remained a trend for a reduction in APC density but this was no longer statistically significant. Similar changes in APC positive oligodendrocyte number were seen in the anterior commissure (S1 Fig).

**Fig 5:**
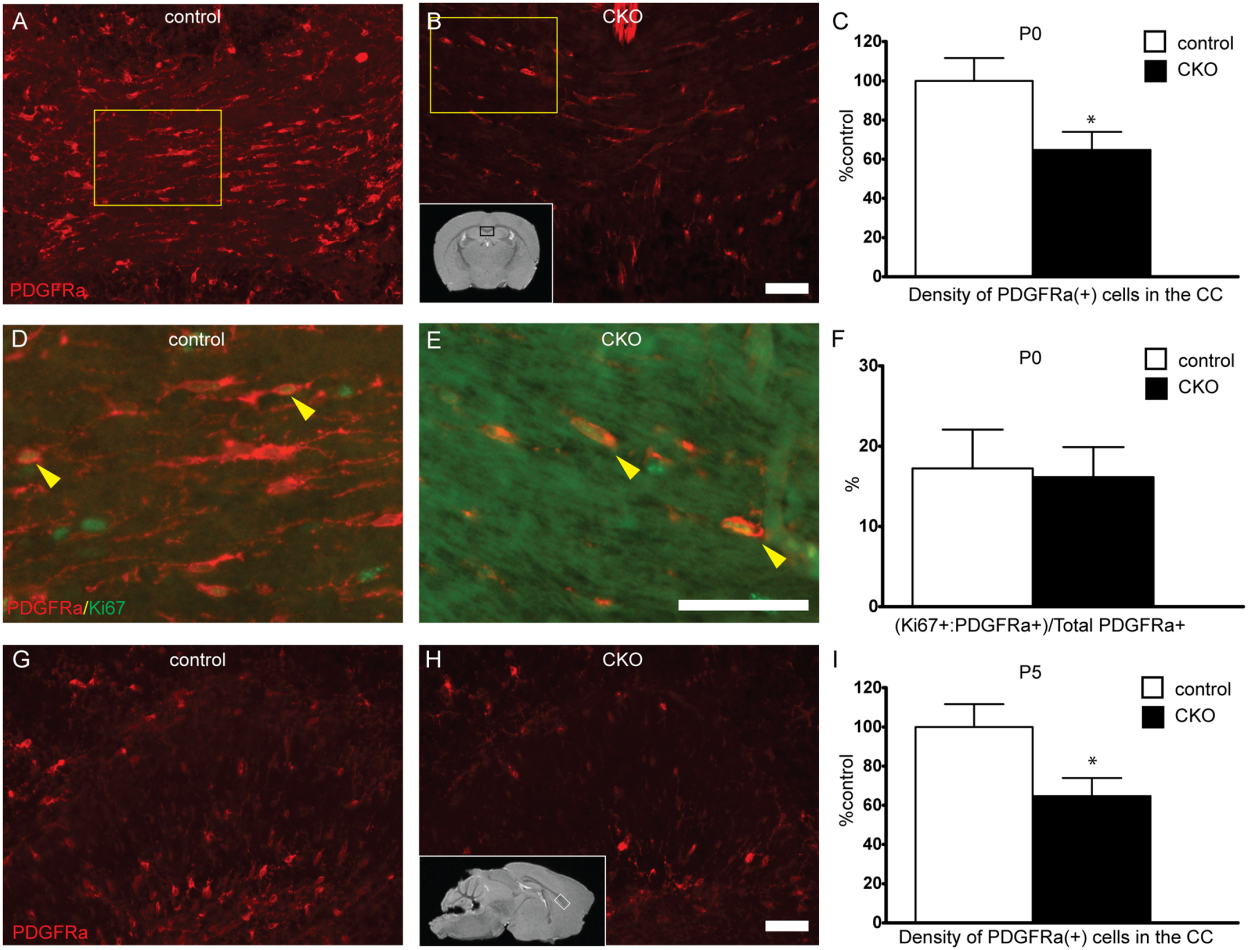
Decreased oligodendrocyte precursor number in developing white matter tracts. To determine if loss of mTORC2 activity alters OPC number, PDGFRα-positive OPCs were counted in the corpus callosum at P0 and P5. (Inset B, black box represents area of CC depicted on coronal sections. Inset H, white box represents area of CC depicted from sagittal sections). To determine if proliferative rate of OPCs was altered following loss of *Rictor*, percent of proliferating OPCs was determined at P0 with co-labeling of PDGFRα and Ki67, a proliferation marker (D-F, yellow box in A and B demonstrate region of interest in D and E, respectively). Data represents mean +/- SEM compared with Student’s t-test, n = 8–10 per group, *p<0.05. Scale bar = 50 μm.

To determine if the reductions in myelination and oligodendrocyte number were due to impaired differentiation of oligodendrocyte precursor cells (OPCs) to oligodendrocytes, PDGFRα positive OPCs were counted in P0 and P5 corpus callosum. Consistent with counts of APC positive oligodendrocytes, OPC numbers were decreased by nearly 40% in CKO animals as early as P0 with a similar reduction present at P5 (Fig 5). Similar findings were seen in the AC (S1 Fig). To determine if the decrease in cell number may be due to alterations in proliferation rate of OPCs, the ratio of Ki67 positive proliferating OPCs was compared between control and CKOs. 16% of PDGFRα positive OPCs were proliferating in the CKO which was not significantly different than the 17% observed in controls. Given the reduction in OPC number at P0, these findings suggests an embryonic role for Rictor in OPC development.

The loss of *Rictor* from either neurons or neuroprogenitor cells in mice has been shown to cause abnormal behaviors, including hyperactivity and decreased markers for anxiety [11,15]. Thus, we hypothesized that hypomyelination may contribute to similar behaviors as those seen upon targeting of neurons. *Rictor CKO* mice demonstrated no gross morphological abnormalities, bred efficiently, and demonstrated comparable body weights to controls throughout development (S2 Fig). No seizures were noted during routine care. Decreased Akt expression has been shown to correlate with brain size[24], thus we questioned whether decreased Akt activation due to loss of mTORC2 activity may result in microcephaly. Neither cortical thickness, isolated brain weight, nor the brain weight to body weight ratio were altered following loss of *Rictor* (control 2.156 ± 0.014% vs CKO 2.051 ± 0.066%, p = 0.1224, n = 5–7 per group).

To determine if hypomyelination due to loss of Rictor from OPCs resulted in a hyperactivity phenotype, we assessed overall activity using open field testing. Total distance explored and the average velocity was unchanged between control and *Rictor* CKO mice (Table 1). Additionally, as anxious mice tend to hold closely to walls, we calculated thigmotaxis, defined as the percentage of distance travelled in the zone along the wall of the box. No differences in thigmotaxis between genotypes were seen.

**Table 1:** Absent behavioral phenotype following loss of Rictor from OPCs.

TEST	control	CKO	p-value (n)
**Open Field**			
Total distance (m)	4.5 ± 0.3	4.5 ± 0.4	0.91 (14)
Average thigmotaxis (%)	48 ± 3	50 ± 2	0.61 (14)
Average velocity (cm/s)	30.6 ± 1.3	30.8 ± 1.3	0.91 (14)
**Elevated zero-maze**			
Distance traveled open (m)	5.8 ± 0.7	6.1 ± 0.4	0.78 (13–15)
Time in open (s)	112 ± 8	122 ± 6	0.34 (13–15)
Duration visit open (s)	7.9 ± 0.7	7.2 ± 0.4	0.40 (13–15)
**Buried Marbles**			
Marbles buried (#)	4.8 ± 0.4	5.0 ± 0.9	0.85 (9–11)
**Rota rod**			
Latency to fall (s)	186 ± 18	195 ± 13	0.69 (9–10)
**Barnes Maze**			
Latency to escape (s)	25 ± 7	21 ± 7	0.69 (9–11)
Distance travelled (m)	8.0 ± 0.5	9.3 ± 0.5	0.11 (9–11)
Velocity (cm/s)	67 ± 4	77 ± 4	0.11 (9–11)

Open field activity was recorded for 50 minutes in a sound-proofed chamber. Total distance, average velocity and thigmotaxis, the percent of time in which the animal stayed near the walls of the chamber, were recorded. To characterize anxiety phenotypes, the elevated zero-maze was utilized with distance traveled in the open regions, time in the open regions and duration of visits to the open regions determined. The buried marble assay characterized compulsive digging as measured by the total number of marbles buried. To characterize motor coordination, Rota rod was used with graded acceleration. n = 13–15 animals per group, p>0.05 by Student’s t-test.

Thin or dysfunctional myelin has the potential to cause functional defects due to reduced axonal conductive properties and impaired communication between the cerebral hemispheres, potentially manifesting in autism relevant behaviors. To evaluate anxiety and obsessive-compulsive-like phenotypes, the elevated zero maze and buried marble tests were utilized, respectively. There were no differences observed in distance traveled, time spent in the open arm, or number of entries into the open arms of the zero maze (Table 1). No significant changes were seen in the number of marbles buried. Lastly, to assess if coordination difficulties could contribute to the lack of an observed behavioral phenotype, Rota rod testing was performed with no differences between genotypes observed.

Finally, to assess spatial learning and memory, mice were trained for 3 days on the Barnes maze and on day 4, latency to finding an escape route, distance travelled and velocity were measured. No significant differences were seen in latency to enter the target zone, distance traveled, or velocity within the maze. Thus, in contrast to *Rictor* loss from neuroprogenitors which resulted in marked hyperactivity and a reduced anxiety phenotype [15], the loss of Rictor from OPCs did not result in an observable behavioral phenotype.

**Fig 6:**
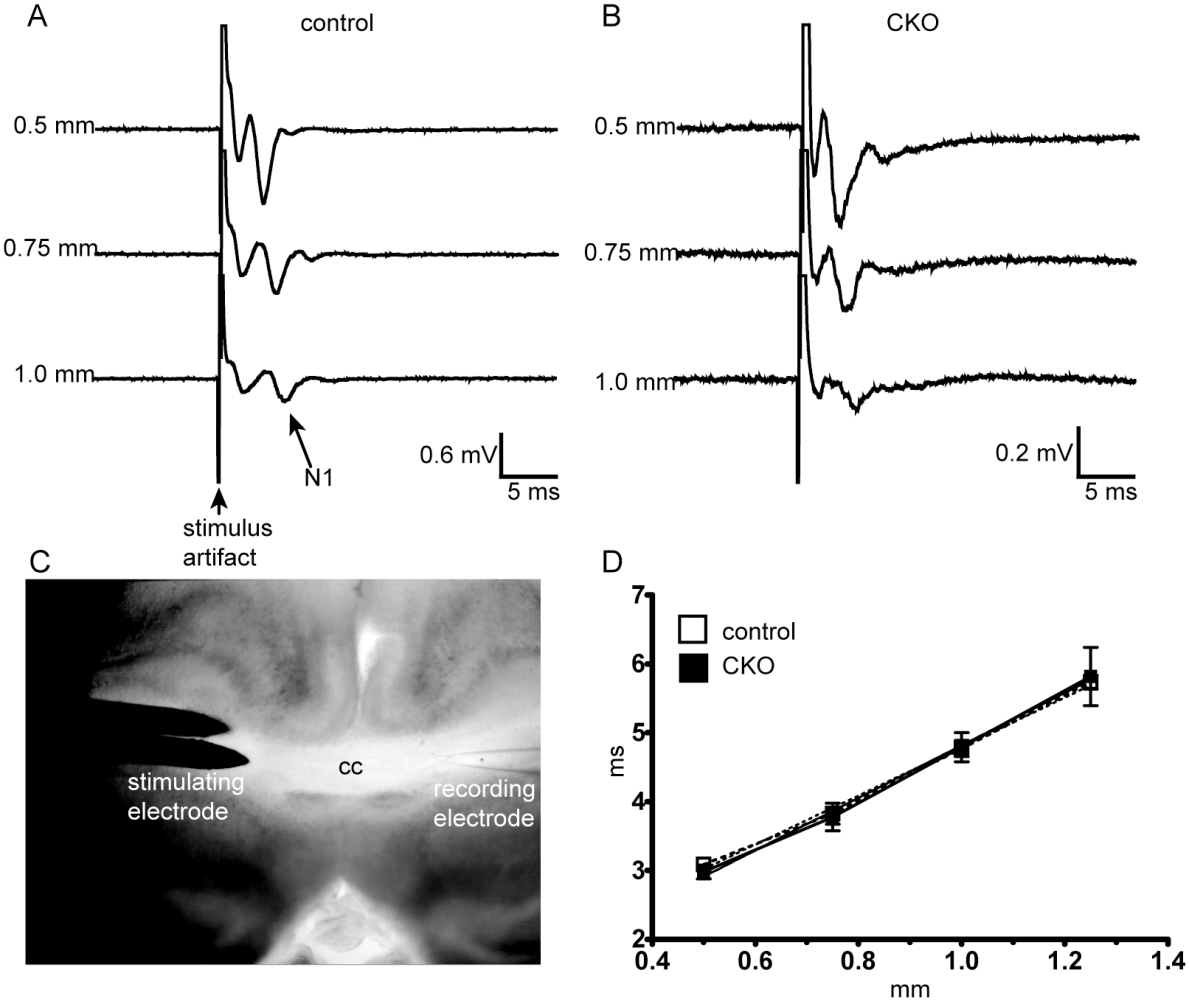
Conduction velocity (CV) across the corpus callosum is not altered in the context of hypomyelination from loss of mTORC2 activity. To characterize conduction, CV was recorded at increasing distances from a recording electrode across the corpus callosum in P32-42 day old mice. (A,B) Representative traces from a control and CKO mouse. (C) Schematic of recording set-up. (D) Plot of velocity against time, from which a conduction velocity was derived. Data compared by linear regression, n = 3 animals per group, F = 0.305, p = 0.5866.

The absence of a clear behavioral phenotype was unexpected, but could be explained if the degree of hypomyelination was not sufficient to alter neuronal function. To address this hypothesis, we measured axonal conduction velocity across the corpus callosum. An N1 signal, representing conduction across myelinated axons (Fig 6), was readily obtained in live brain slices from control and CKO mice. The conduction velocity was essentially identical in both the control and CKO groups.

**Fig 7:**
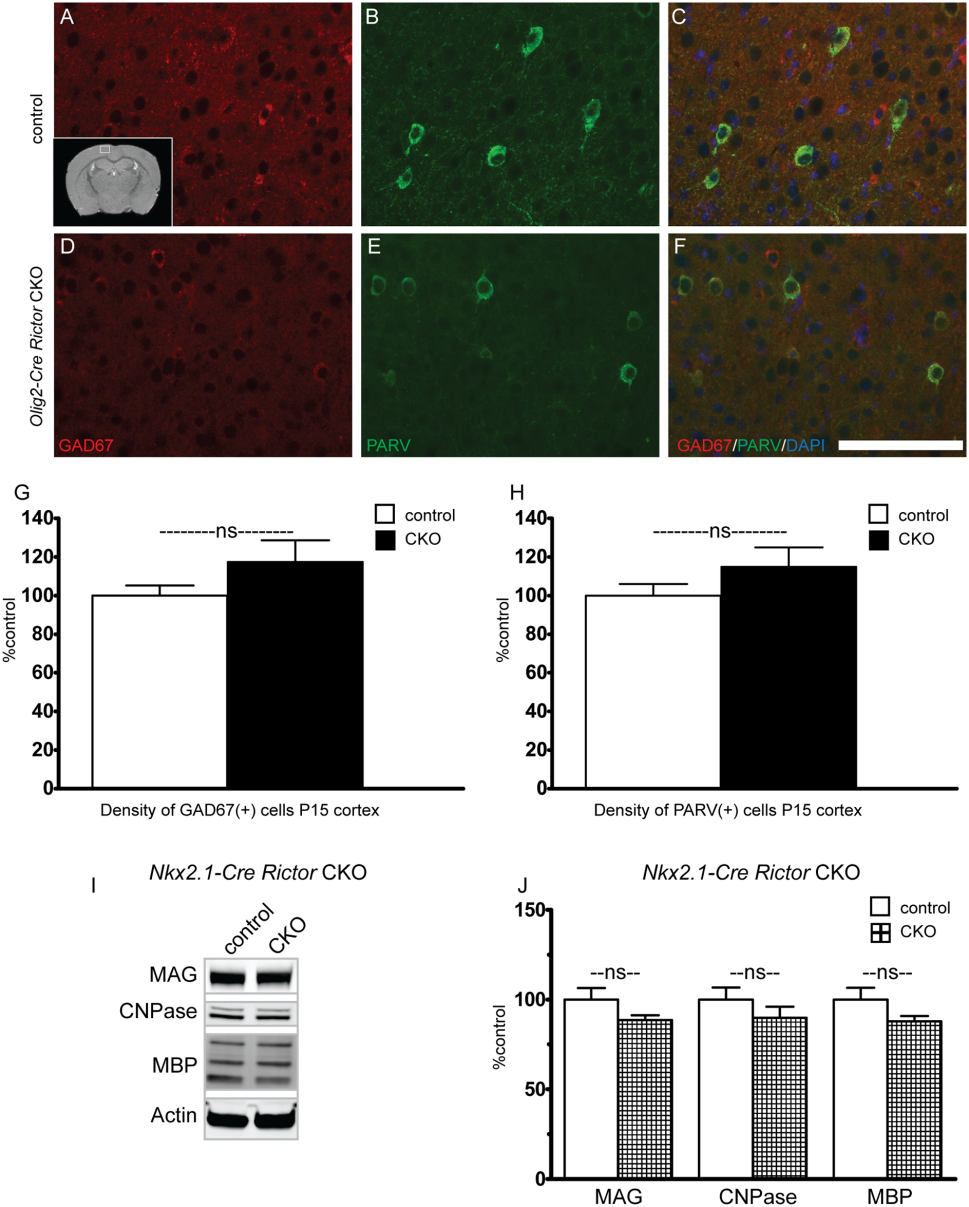
Loss of Rictor from GABAergic interneurons does not alter interneuron number or contribute to hypomyelination. To determine if loss of Rictor from GABAergic interneurons may alter interneuron proliferation, the number of parvalbumin and GAD67 expressing interneurons was determined in control (A-C, G-H) and CKO (D-H) cortex (Inset A, while box denotes location of images). Data represents mean +/- SEM compared with Student’s t-test, n = 6–7 per group, *p<0.05. Scale bar = 200 μm. To determine if loss of Rictor from GABAergic interneurons may contribute to hypomyelination, *Rictor* was inactivated in interneuron progenitors using *Nkx2*.*1-Cre*. Expression of the myelin associated proteins MBP, CNPase and MAG was determined in P30-P60 cortex. Data represent mean +/- SEM compared with Student’s t-test, n = 4 animals per group.*p<0.05.

During development, Olig2 is involved in the specification of a variety of cell types, including interneurons[25]. As interneurons are known to play a role in oligodendrocyte development and migration, we sought to determine the relative contribution of *Rictor* loss in interneurons to the observed hypomyelination phenotype. Cortical parvalbumin and GAD67 positive interneurons were counted at P15, a time at which both expression of myelin proteins and oligodendrocyte number was markedly reduced in the *Olig2-Rictor* CKO. Unlike OPC and oligodendrocyte numbers, interneuron number was not significantly altered in the CKO cortex (Fig 7). Additionally, we analyzed myelin protein expression in *Nkx2*.*1-Rictor CKO mice*[26]. Between P30-P60, a time in which the *Olig2-Rictor* CKO animals demonstrated approximately 40–70% reductions in expression of myelin proteins (Fig 1), no significant reduction in expression of myelin proteins was seen in the *Nkx2*.*1-Rictor* CKO. These data suggest that cell autonomous loss of *Rictor* in oligodendrocytes is the key contributor to hypomyelination, while *Rictor* in interneurons does not support a significant role in myelination.

## Discussion

During the past decade, a myriad of signaling pathways have been found to converge on the mTOR kinase. While mTORC1 signaling in the CNS has been well characterized, the role of Rictor and the mTORC2 complex has received much less scrutiny. As mTORC2 signaling is reduced in animal models of TSC, likely due to feedback inhibition, we hypothesized that loss of mTORC2 activity contributes to the disease phenotypes seen in TSC. We previously demonstrated marked behavioral alterations and hypomyelination resulting from loss of *Rictor* in neuroprogenitor cells, including a subset of OPCs[15]. Utilizing a mouse model that results in loss of *Rictor* in oligodendrocyte precursor cells, we now demonstrate that loss of *Rictor* in OPCs is sufficient to cause a developmental hypomyelination.

Our data demonstrate a mild hypomyelination phenotype following loss of *Rictor* in oligodendrocyte precursor cells, consistent with the findings of Bercury et al. who targeted mature oligodendrocytes[13]. Ultrastructural analysis of myelination over time demonstrated a significantly reduced g-ratio, myelinated axon density, and myelin fraction at P30 in the CKO which was nearly normalized by P60. These data suggest that the overall decrease in myelin content represents a decrease in the number of myelinated axons versus a dramatic decrease in the amount of myelin per axon. The increase in g-ratio at P30 is likely due to preferential myelination of larger caliber axons in the CKO.

To determine the etiology for this reduction in myelin content, we counted both OPCs and oligodendrocytes, noting a significant reduction in numbers of both OPCs and mature oligodendrocytes from white matter tracts. The relative degree of hypomyelination did improve over time, commensurate with a trend towards an increased density of mature oligodendrocytes. While the overall expression levels of proteins are similar between our study and that reported by Bercury et al., subtle differences may be related to technique (we included CC with cortex), selection of Cre driver and time points studied. One clear difference is that Olig2 is expressed very early in oligodendrocyte development whereas CNPase is expressed at a later developmental stage. The proportional reduction of PDGFRα positive OPCs seen at P0 and P5 with APC positive oligodendrocytes at P15 is consistent with the observation of Bercury et al. that oligodendrocyte maturation is not affected by loss of *Rictor*. Additionally, as post-natal proliferation was not different in the CKO, this suggests an embryonic role for *Rictor* in OPC generation, though whether the embryonic proliferation rate is reduced is not clear. The improvement in myelination over time may be due in part to population of the cortex by cortically derived OPCs which were not specified by *Olig2-Cre* [27].

As *Olig2* plays a role in specification of a variety of cell types during normal development [28], altered embryonic specification may both decrease OPC numbers and alter expression of other cell types, including interneurons. Given the possibility that targeting of interneurons may potentially confound our findings, we determined that interneuron number, unlike OPC number, is not significantly reduced in the *Olig2-Cre Rictor* CKO. Further, we determined that expression of myelin proteins is not significantly altered upon preferential targeting of *Rictor* in interneurons using *Nkx2*.*1-Cre*. These data suggest that the observed myelination changes in *Olig2-Rictor* CKO mice are not due to non-cell autonomous effects of *Rictor* inactivation in interneurons.

Abnormalities in myelination are increasingly being associated with neurodevelopmental diseases, including cryptogenic autism and autism related to TSC [2,29]. We hypothesized that the altered myelination following loss of *Rictor* may contribute to neurobehavioral sequelae. Loss of Rictor from neurons has been shown to alter prepulse inhibition and the dopamine to norepinephrine ratio[11]. We previously reported hyperactivity and reduced anxiety in the *Emx1-Rictor CKO* mice[15] in which neural progenitor cells (including oligodendrocyte and astrocyte precursors), were targeted. In contrast, the adult *Olig2-Rictor* CKO, did not demonstrate differences in activity, spatial memory, or anxiety phenotypes from the control animals. These findings are not unexpected in context of the mild hypomyelination and unaltered conduction velocity. The extent of dysmyelination by P60 was not sufficient to alter the myelin’s resistive properties, thus preserving conduction velocity down the axon. Additionally, as conduction velocity also depends on axonal radius to the fourth power, the slight increase in axon diameter seen in the genu and splenium of the CKO[18] may represent a compensatory mechanism which maintains normal velocity[30].

As noted above, the underlying mechanism for the reduction in OPC number is not entirely clear and may be due in part to decreased progenitor proliferation or decreased OPC specification during embryogenesis. As we also reported decreased oligodendrocyte number after loss of *Tsc2*[9], we hypothesize that decreased Akt activity and an mTORC1 independent process is contributing to the decrease in oligodendrocyte number. Bcl-2 associated death promoter (BAD) and GSK3β are both negatively regulated by Akt, thus decreased Akt activity may lead to increased apoptosis or reduced cell proliferation, respectively, and subsequent reductions in OPC numbers[31,32].

Our findings reinforce and expand on published data demonstrating a contributing role for mTORC2 activity in myelination[13]. In contrast to severe and sustained hypomyelination caused by attenuated mTORC1 activity, the role of mTORC2 activity in myelination is significant, but minor compared to mTORC1. We postulated that decreased mTORC2 activity may significantly contribute to the hypomyelination observed following loss of *Tsc2*, though the stark differences in the magnitude of the hypomyelination between the *Olig2-Rictor* and *Olig2-Tsc2* models suggests that mTORC1 over activation is the major contributor to hypomyelination in the *Olig2-Tsc2* model. Lastly, the absence of a behavioral phenotype in *Olig2-Rictor* CKO mice and the marked phenotypes in the neuroprogenitor *Emx1-Rictor* CKO and the neuronal *Nestin-Rictor* CKO mice [11] suggest a pervasive role for mTORC2 activity in neuronal function versus a transient role in oligodendrocyte development.

## Supporting information

S1 Fig Decreased mature oligodendrocytes and OPCs in the anterior commissure following loss of *Rictor*. To determine if loss of mTORC2 activity alters oligodendrocyte number, numbers of APC-positive mature oligodendrocytes were counted in the anterior commissure in P15 (A, D, G), P60 (B, E, G) and P104-P111 (C, F, G) day old mice. (Inset A, black box represents area of AC depicted on sagittal sections). To determine if loss of mTORC2 activity alters OPC number, PDGFRα-positive OPCs were counted in the anterior commissure at P5. Data represents mean +/- SEM compared with Student’s t-test, n = 4–10 per group, *p<0.05, **p<0.01. Scale bar = 200 μm.
(TIF)

S2 Fig Brain and body size are not altered following loss of mTORC2 in oligodendrocytes. No significant differences in animal weights were noted during development (A-B). In a cohort of adult female mice used for MRI imaging at P60-P70, no significant differences were noted in body or brain weight. Cortical thickness as measured from high resolution T_1_-weighted MRI images was similar between groups. Data represent mean +/- SEM compared with Student’s t-test, n = 5–7 animals per group.
(TIF)
