# Supplementary figures and images for: Loss of mTORC2 signaling in oligodendrocyte precursor cells delays myelination
Source: PLoS One. 2017 Nov 21;12(11):e0188417. doi: 10.1371/journal.pone.0188417 (PMC5697806; doi:10.1371/journal.pone.0188417)

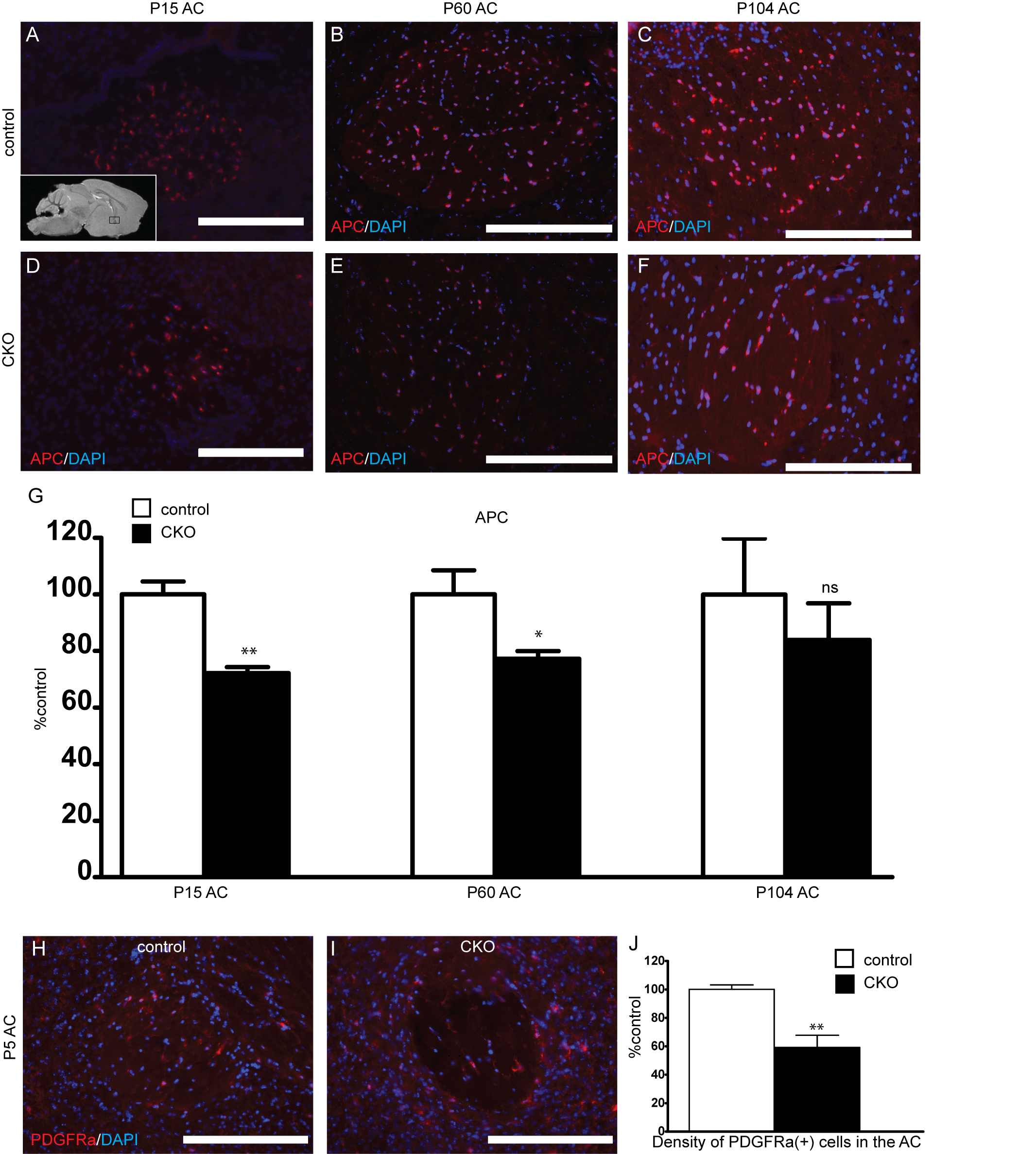

Supplement: S1 Fig — To determine if loss of mTORC2 activity alters oligodendrocyte number, numbers of APC-positive mature oligodendrocytes were counted in the anterior commissure in P15 (A, D, G), P60 (B, E, G) and P104-P111 (C, F, G) day old mice. (Inset A, black box represents area of AC depicted on sagittal sections). To determine if loss of mTORC2 activity alters OPC number, PDGFRα-positive OPCs were counted in the anterior commissure at P5. Data represents mean +/- SEM compared with Student’s t-test, n = 4–10 per group, *p<0.05, **p<0.01. Scale bar = 200 μm. (TIF) [file pone.0188417.s001.tif]

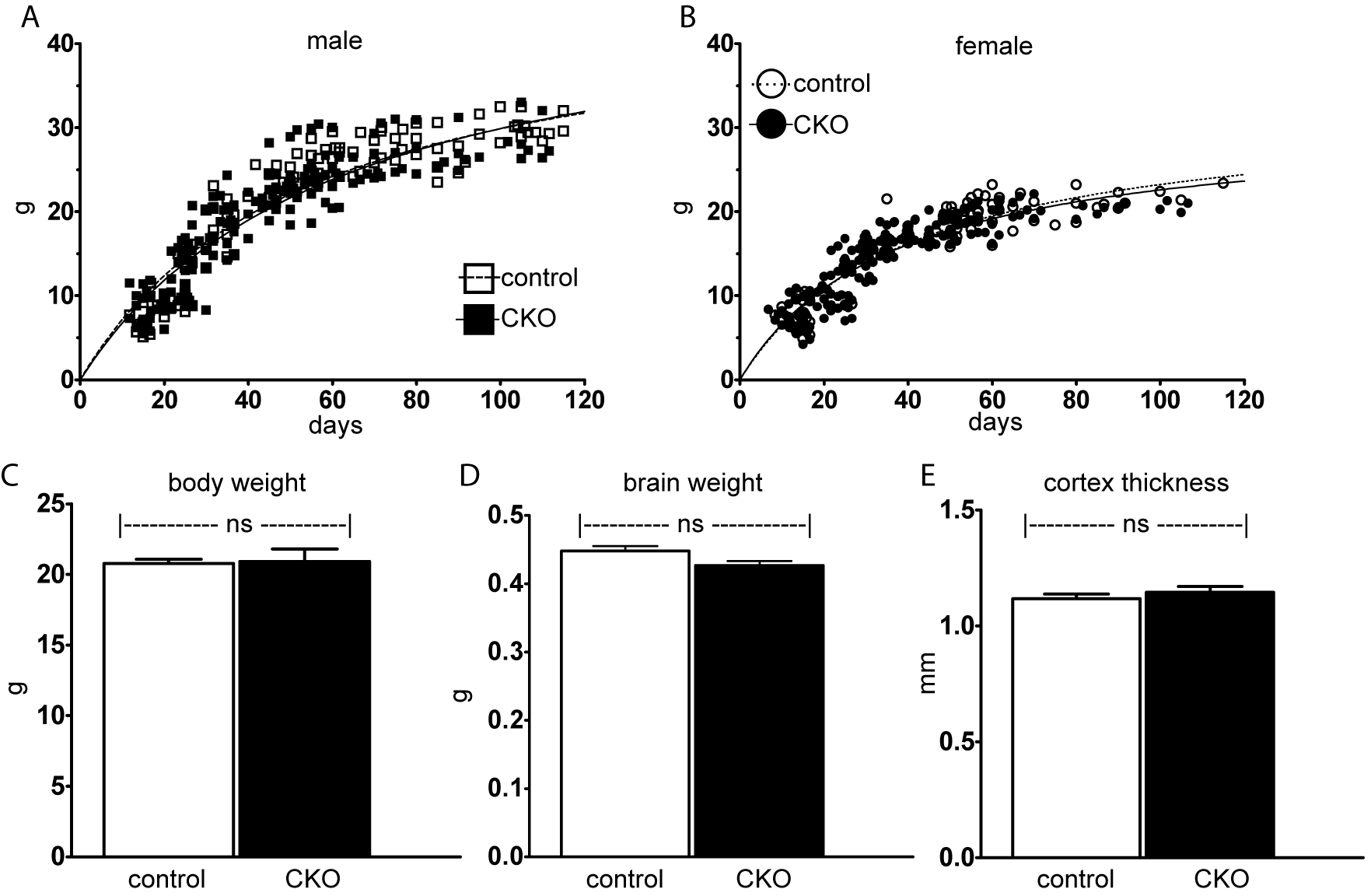

Supplement: S2 Fig — No significant differences in animal weights were noted during development (A-B). In a cohort of adult female mice used for MRI imaging at P60-P70, no significant differences were noted in body or brain weight. Cortical thickness as measured from high resolution T1-weighted MRI images was similar between groups. Data represent mean +/- SEM compared with Student’s t-test, n = 5–7 animals per group. (TIF) [file pone.0188417.s002.tif]
